# Supplementary material for: Risk of New Primary Cancer in Patients with Posterior Uveal Melanoma: A National Cohort Study
Source: Cancers (Basel). 2022 Jan 7;14(2):284. doi: 10.3390/cancers14020284 (PMC8773914; doi:10.3390/cancers14020284)
Supplement: Supplementary file 1 [file cancers-14-00284-s001.zip › cancers-1511053-supplementary.pdf]

cancers MDPI

Table S1. Hazard ratio HR(t) with 95% confidence intervals (CI) for incidence of new primary cancer in patients with posterior uveal melanoma (UM) compared to unexposed comparisons. The model was adjusted for gender, age at diagnosis/index date, calendar year of diagnosis, disposable income levels, civil relation and education.

	Level	HR	95% CI	p-value
Diagnosis of Posterior UM		1.37	(1.20; 1.55)	0.001
Gender (ref: Male)	Female	0.70	(0.65; 0.75)	<0.001
Year of diagnosis/index date (ref: 2010-2016)	1980-1989	0.86	(0.74; 1.00)	<0.044
	1990-1999	0.89	(0.78; 1.02)	0.084
	2000-2009	0.98	(0.86; 1.11)	0.75
Age (years) (ref: 60-69)	<20	0.15	(0.038; 0.61)	0.008
	20-39	0.16	(0.13; 0.20)	<0.001
	40-49	0.42	(0.37; 0.48)	<0.001
	50-59	0.61	(0.55; 0.67)	<0.001
	70-79	1.39	(1.27; 1.53)	<0.001
	>80	1.46	(1.24; 1.72)	<0.001
Civil relation	In relationship	0.98	(0.91; 1.059)	0.65
Income (ref: level 1)	2	1.002	(0.90; 1.12)	0.97
	3	0.99	(0.89; 1.11)	0.90
	4	0.97	(0.87; 1.086)	0.64
	5	0.99	(0.89; 1.11)	0.91
Education duration (ref= Long)	medium	1.022	(0.92; 1.37)	0.69
	Short	0.99	(0.89; 1.11)	0.88
	Unknown	0.86	(0.75; 1.00)	0.047
